# Beliefs about the controllability of social characteristics and children’s jealous responses to outsiders’ interference in friendship

**DOI:** 10.1371/journal.pone.0209845

**Published:** 2019-01-16

**Authors:** Kristen L. Lavallee, Jeffrey G. Parker

**Affiliations:** 1 Ruhr-University Bochum, Bochum, Germany; 2 The University of Alabama, Tuscaloosa, Alabama, United States of America; Middlesex University, UNITED KINGDOM

## Abstract

Although some jealous children respond to outsider interference in friendships with problem solving and discussion, others withdraw from the relationship or retaliate against the friends or others. Beliefs about the nature of social characteristics are proposed as an explanation for behavioral heterogeneity in response to jealous provocation. Based on learned helplessness theory and research on children’s implicit personality theories, children who subscribed strongly to the belief that social characteristics are fixed and that social outcomes are uncontrollable (high entity beliefs), were expected to more strongly endorse asocial and antisocial responses and less strongly endorse prosocial responses to outsider interference than children who did not have strong entity beliefs, depending on their internal versus external attributions of blame. Two hundred eighty-six children in sixth through eighth grades (primarily Caucasian) participated in an experimental test of this hypothesis. Although hypothesized interactions between beliefs and locus of blame were not supported, results indicated that children who believe social characteristics are changeable also believed they had more control in the internal condition than children who believe social characteristics are immutable. Further, pessimistic children were more likely to tend to endorse asocial and antisocial behavior and less likely to endorse prosocial behavior than optimistic children.

## Introduction

Children and adolescents show considerable heterogeneity in their emotional and behavioral responses to their friends’ other friends and extradyadic social activities [1]. Lavallee and Parker [2] for example, report small to moderate positive correlations between feelings of jealousy in these circumstances and several negative behaviors, including friend surveillance, rifling through a friend’s belongings in search of evidence, interrupting a friend who is talking to someone else, and engaging in certain forms of aggression, such as trying to get other children to exclude a certain individual from the group. These and other coercive or aggressive responses to jealousy may increase conflict with friends [3], and ultimately contribute to relationship dissolution and mutual antipathy [4]. However, jealousy apparently also sometimes increases self-reported positive behaviors, such as voicing one’s feelings, and making attempts to include everyone in subsequent activities [5]. A better understanding of children’s responses to jealousy is essential to understanding the impact of jealousy on children’s friendships, and may be partially illuminated via an understanding of the cognitions accompanying children’s jealous feelings.

### Perceptions of control and implicit theories

Children’s social cognitions play an important role in their interpretation of social situations, as well as selecting and guiding behavioral responses to others’ actions [6]. Much research has illuminated the role of attributional style for negative events, and its impact on adjustment as well as behavioral response. Children interpret events in light of their attributions for causality and control, in the domains of locus of control (i.e., whether the cause is internal or external), stability (whether the cause is stable over time or transient), globality (whether the cause affects only a small area of life, or spans many areas), and controllability (whether the cause is subject to change or is fixed). Such attributional styles are reliable predictors of social, emotional, and academic adjustment [7–10]. According to helplessness theory [7], attributing negative events to stable, internal, and global sources comprises a pessimistic explanatory style, that leads to greater adjustment difficulty, whereas attributing negative events to unstable, external, and specific causes comprises a more optimistic style that leads to greater perceptions of the events as malleable and changeable, and thus leads to greater emotional and behavioral adjustment. Individuals who tend to view events as uncontrollable do not expect their behavior to have an impact on future outcomes, and thus give up more easily when faced with challenges, demonstrating a helpless orientation to behavior across multiple domains, leading to increases in depression and other difficulties in children (e.g., [11, 12]).

Individuals’ locus of control and beliefs about how much they can or cannot control certain situations may be factors in behavioral responses to perceived problems within a friendship, yet are under-explored in the domain of friendship interference. The tendency to hold certain control beliefs across events, is influenced by children’s broader implicit assumptions concerning the malleability of personal qualities and traits [13–15]. In a now classic experiment in the academic domain, students presented with entity theories (i.e., that intelligence is fixed, and failure is due to lack of ability) were less likely than those with incremental theories (i.e., intelligence is malleable and can thus be changed through effort) to use remedial strategies to improve their skills when they performed poorly [14]. Presumably as a result of increased effort, individuals who believe they have control over their achievement improve in self-efficacy and performance over time when compared to entity theorists [16]. Further, teachers who emphasize performance and point out ability differences between students, rather than emphasizing mastery, individual learning, and effort, foster a sense of helplessness in students, and devaluation of academic achievement [17]. Entity and incremental beliefs may also affect social behavior, via their impact on attributions of blame and their beliefs about the controllability of social situations.

Over the past two decades, the construct of implicit theories, developed initially by Dweck and Leggett [18], has matured and grown in visibility and utility [19], and expanded into the social domain. Dweck and colleagues define implicit theories as core assumptions about the malleability of personal traits and abilities and suggest that individual children tend to fall in one of two contrasting patterns. So-called incremental theorists adhere to the view that personal traits are inherently malleable and thus changeable through effort. In contrast, entity theorists believe that human attributes are fixed and difficult or impossible to change. Children who believe social skills and personality are fixed traits may be discouraged from making further attempts at social interaction after experiencing social setbacks, and may withdraw from social situations or employ face-saving defensive strategies to cope with perceived rejection. Graham and Juvonen [20, 21] demonstrated that children who attributed their victimization by peers to their stable personality or character traits (characterological self-blame) were less likely to change their behavior and more likely to be chronically victimized. On the other hand, children who attributed their victimized to their behavior (behavioral self-blame) took action to change negative or annoying behaviors and, as a result, were less likely to be chronically victimized and better adjusted. In another study, children who were given a performance goal (i.e., to demonstrate their fixed ability) were less likely than children given a learning goal (i.e., to develop their flexible ability) to try new strategies in writing effective follow-up letters after their initial rejection from a pen-pal club [22]. Further, entity theorists wrote shorter follow-up letters, and expressed fewer positive feelings in their letters, than incremental theorists, regardless of goal condition. Erdley and Dweck [23] found that children who held an entity view of others were more likely to persist in their stereotypes about others, even when presented with counter-evidence, and were less empathetic and more likely to recommend punishment for negative behaviors than children who held a view of others as changeable. Others have also found that entity theorists were more punitive, whereas incremental theorists recommended remediation for transgressions, such as cheating on a test or dating someone else’s girlfriend [18, 24].

Likewise, it appears that entity theorists are more likely than incremental theorists to endorse retaliation (such as aggression) in response to hypothetical circumstances of personal victimization (e.g., their partner leaves the relationship without explaining why), whereas incremental theorists are more likely to attempt to understand the other person and forgive, talk to, or educate them [18, 24]. Whether the effects of beliefs on responses to hypothetical situations translate into real world behavior is still unknown. In another study, adult shy incremental theorists engaged in more approach and less avoidance of social situations than shy entity theories, in both self-report and observational coding [25]. Additionally, research with adults indicates that entity theories of emotion are associated with lowered emotion regulation, and with negative social and emotional outcomes [26].

This research suggests the importance of incremental and entity theories, resulting perceptions of control, and attributions of locus of control (internal or external) in predicting social behavior in the case of children’s close friendships. In a study of sociometric status, one indicator of success in the social milieu, Earn and Sobol [27] found that popular children were more likely than children in other sociometric groups to attribute hypothetical social failures and successes to controllable causes, suggesting that incremental theorists would be more likely to modify behavior to makes them more well-liked (or vice versa). In a similar study, popular children were less likely than controversial, neglected, or rejected children to use “luck” as an explanation for social events [28], suggesting that their beliefs affect their behavior, which in turn affects their likeability by peers. The reverse order of effects is also possible. Control beliefs and social behavior in children is an area in need of more research in general. In the field of responses to friendship interference, the role of such cognitions on behavior is entirely unexplored.

### The present study

The broad goal of the present study is to illuminate a set of cognitive conditions under which individuals perceive control over the situation, and endorse adaptive versus maladaptive behavioral responses to perceptions of friendship threat due to interlopers, using assessments of children’s implicit theories, as well as a within-subjects experimental manipulation of locus of control. Specifically, children who view their own and others’ social characteristics as changeable were hypothesized to engage in more reparative, prosocial overtures, such as talking to their friend, making attempts to include everyone, or trying other forms of problem solving. In contrast, children who believe their social characteristics are fixed were predicted to give up or to use asocial strategies to deal with being left out by a friend, especially when participants perceive that the interference is their own fault (i.e., make an internal attribution). Finally, children who view others’ social characteristics as fixed and attribute the root of the problem to the friend (i.e., make an external attribution) were expected to engage in more anti-social behavior such as aggressing or retaliating against their friend than those who view social characteristics as malleable. Entity theorists were expected to believe that the problem is stable and global and as a result assume that they have little control over the outcome of the jealousy-invoking situation. Because entity theorists in the external condition also believe they have been slighted (i.e., they make an external attribution), they were expected to be motivated to save face or to punish the partner. Effects were expected to be amplified in children who experience stronger feelings of jealousy.

Specifically, we test the following hypotheses:

1. Self-and other entity beliefs would interact with condition to influence controllability beliefs following the internal/external manipulation. That is, those on the entity rather than the incremental end of the entity beliefs spectrum would be more likely to report thinking they can do something about the events in the vignette, especially when the vignette depicted negative friendship events as one’s own responsibility (external condition).
2. Children with high characteristic jealousy will engage in more behavioral responses of any type, than children with generally lower levels of jealousy.
3. Children will generally engage in more prosocial than asocial and antisocial responses.
4. Females would generally endorse more prosocial, and males more asocial responses, but that jealousy would be a stronger predictor of behavioral responding than sex.
5. Children would be more likely to endorse prosocial responses in the own-fault (internal control) condition and asocial or antisocial responses in the other-fault (external control) condition.
6. Those with high self-relevant entity beliefs in the internal control condition and with high jealousy would engage in more prosocial behaviors in response to friendship interference.
7. Pessimistic explanatory style may account for the effect of entity beliefs on behavior.
8. Those with high other-relevant entity beliefs in the external control condition and with high jealousy would engage in more antisocial behaviors in response to friendship interference.

## Method

### Participants and procedure

Two hundred eighty-five students from a rural middle school participated (33 girls, 46 boys in the 6^th^ grade, 53 girls, 55 boys in the 7^th^ grade, 55 girls, 43 boys in the 8^th^ grade). The school’s total enrollment was 643. Five hundred ninety-one students agreed to participate but 305 of those were randomly selected (by classroom) to do for time considerations. Participants were 95.8% Caucasian-American, .3% Asian, 1% other, and 2.9% unknown. Following the protocol approved by the Institutional Review Board of the Office for Research Protection at the Pennsylvania State University, families of potential participants received letters detailing the study and soliciting parental consent and research staff visited classrooms to describe the study to the students. Both students and their parents gave written informed consent to participate. As an incentive for returning consent forms (regardless of the decision to participate), students in classrooms with greater than a 90% return rate were entered into a raffle for a $20 gift certificate to a local store. Assessments were obtained for the entire sample during group testing in their classrooms on two occasions in the spring. Each session was approximately 45 minutes in length.

### Measures

#### Entity views of social characteristics

Children’s beliefs that their own and others’ social characteristics are fixed rather than malleable were assessed using six items adapted from Dweck and colleagues to fit the social domain [14, 22]. Participants indicated agreement on a scale from 1 (strongly disagree) to 5 (strongly agree). Three items referred to the participants’ own social characteristics (e.g., “Whether you are loyal and trustworthy as a friend is something that is deeply ingrained into your personality. It can’t really be changed.”), and the remaining three items referred to others’ social characteristics (e.g., “Whether someone is loyal and trustworthy as a friend is deeply ingrained into their personality. It can’t really be changed.”). Items were adapted from the original scales by altering the context from the academic to social domain. Internal consistency of the three self-referent items was .67 and the consistency of the three other-referent items was .71. Although self and other entity views were significantly positively correlated, *r* = .59, p < .001, they were not combined for the analyses due to their conceptual differences and the possibility that entity views may affect behavior differently depending on whether they are relevant to the self or other.

#### General pessimistic explanatory style

The Children’s Explanatory Style Questionnaire [29] was used to assess children’s general tendency to explain negative events in an optimistic versus pessimistic way. Children read hypothetical negative events and selected one of two alternative explanations for why the event occurred. Response options vary systematically along one of three explanatory dimensions: internal versus external (8 items), stable versus unstable (8 items) and global versus situation specific (8 items). In the present study, scores for the stable and global items only were averaged to create a composite pessimism scale (α = .61).

#### Vulnerability to jealousy

Children’s characteristic vulnerability to jealousy was assessed using a subset of ten items from the 27-item Friendship Jealousy Questionnaire [30]. Children read vignettes depicting hypothetical social situations where their best friend and a third party appear to be getting along well or engaging in social activities without them, and then indicated how jealous they would feel under such circumstances from 0 (“Not at all true of me”) to 4 (“Very true of me”). Responses were averaged across items, and were highly internally consistent, alpha = .91.

### Attributions and behavioral responses to outsider interference

#### Stimulus vignettes

Children read four hypothetical stories involving the participant, his or her best friend, and a third party interloper. During the course of each story, the participant looks forward to engaging in an attractive activity with his or her best friend but later learns that the best friend attends with someone else These stories capture a range of realistic events including some that represent one-time events or missed opportunities and others that portray recurring events that may be correctable in the future. Pilot studies and previous research (Roth, 2002). indicate that these vignettes provoke strong feelings of jealousy in most adolescents and young college students. Internal versus external attributions were manipulated and counterbalanced across vignettes (e.g., the friend’s behavior is or is not the participant’s fault), within subjects. Apart from suggesting that the participant did or did not contribute to the friend’s decision to participate in an activity with someone else, no other details were provided concerning why the friend behaved in this way. That is, although participants were told in some cases that the friend’s behavior was angry reaction to something they did, they were not told what specifically triggered this reaction by the friend to avoid altering participant’s responses by their conclusions concerning whether the triggering event was within or beyond their control. The order of the internal versus external attribution manipulation was counterbalanced across vignettes. A version of the story involving a female participant with internal control is as follows:

Imagine that you and your best friend have gone on a weekend skiing trip with her family every winter for the last five years. Last year, you and your best friend had a lot of fun and you can't wait to go again this year. The week before the trip, you are out shopping at a sporting goods store for ski equipment for the trip. At the store you spot another girl, Maxine, trying on a ski jacket and goggles. Maxine and your best friend know each other from their soccer team. You go over to her and say, "Hey Maxine! Shopping for ski equipment?" She says that she needs to get some new ski gear because your best friend invited her to go skiing with her and her family next week. You suddenly remember something that happened earlier, and now you realize this is your fault!

#### Manipulation check

Following each vignette, participants were queried to insure they recognized the manipulation in the assignment of blame from the self (internal) to the best friend (external).

#### Controllability

Finally, after each jealousy-invoking vignette children rated their perceptions of control over the course of their friendship using two items based on Lepore and et al. [31]. Responses were indicated on a scale ranging from 1(disagree a lot) to 5 (agree a lot) and items were averaged within and across vignettes in each condition (alpha is .77 across the internal vignettes, and .74 across the external vignettes).

#### Behavioral responses

After reading each vignette, participants indicated on a Likert scale ranging from 1 (Not likely) to 5 (Very likely) the likelihood that they would engage in each of nine varying responses using items adapted from prior research (i.e., [2, 5, 32]. Response options were chosen to reflect three broad domains: Asocial (withdrawal, dismiss, and ruminate), Antisocial (relational aggression, direct aggression, and passive aggression), and Prosocial (constructive verbal communication, attempts to include all, and attempts to restore the relationship through compensation and making oneself attractive to the partner. Internal consistencies and inter-item correlations within and across scales suggested the need to re-conceptualize the placement of the rumination item as it was strongly positively correlated with all three prosocial items. It seems to thus represent a form of prosocial response focusing on the relationship and the reasons surrounding relationship difficulty and was included in the prosocial rather than asocial scale. Internal consistencies for the final scales for each of the two conditions were all adequate: for asocial: 64 (internal) and .61 (external); for antisocial, .89 (internal) and .88 (external), and for prosocial, was .89 for both the internal and external conditions.

## Results

### Preliminary analyses

#### Bivariate correlations and means

Table 1 presents the means, standard deviations, and bivariate correlations among the measures of entity beliefs, jealousy, and general pessimism for each sex. As shown, self and other entity beliefs were significantly positively related to general pessimism among girls but not among boys. In contrast, neither self- or other-relevant entity beliefs were significantly related to grade or characteristic jealousy, for either boys or girls.

**Table 1:** Correlations and means for self-report measures.

	Grade	Entity self	Entity other	Jealousy	Pessimism
Correlations					
Grade		-.05	-.03	.05	.18*
Entity self	.12		.68**	-.07	.26**
Entity other	.03	.48**		-.02	.21*
Jealousy	-.15	-.04	-.07		-.02
Pessimism	.09	.06	.10	-.07	
Means (SD)					
Females	7.16 (.78)	2.72 (1.09)	2.91 (1.01)	1.57 (.86)	1.28 (.16)
Males	6.97 (.79)	2.82 (1.95)	2.91 (.87)	0.91 (.73)	1.30 (.16)

Note.

* = *p* < .05

** = *p* < .01. Girls are above the diagonal, boys below. N = 137–141.

T-tests comparing males and females on each variable indicated that females reported higher jealousy than males, *t*(1, 277) = 6.91, *p* < .001, *d* = .83, and males reported higher direct aggression than females, *t*(1, 277) = 3.67, p < .001, *d* = .55. Males and females did not differ on the other variables.

#### Manipulation check

Frequencies were calculated to determine the percent of participants who correctly identified the source of blame in the external versus internal conditions. Ninety percent of participants recognized that the situation was not their fault in the external condition, and 57% of participants recognized that the situation was their fault in the internal condition.

### Entity beliefs and controllability

Two 2 (sex) X 2 (high versus low entity beliefs; median split) X 2 (internal versus external condition) repeated measures ANCOVAs were conducted to examine the influence of entity beliefs and condition on controllability beliefs following the vignettes. In the ANCOVA, children’s self-referent entity beliefs were examined in relation to controllability attributions. In the second, children’s other-referent entity beliefs were used in place of self-referent beliefs. In these analyses, general pessimism was included as a covariate to control for the general tendency to think fatalistically about negative events. Condition served as a within subject variable in these analyses and sex and entity beliefs served as between subjects factors.

**Fig 1:**
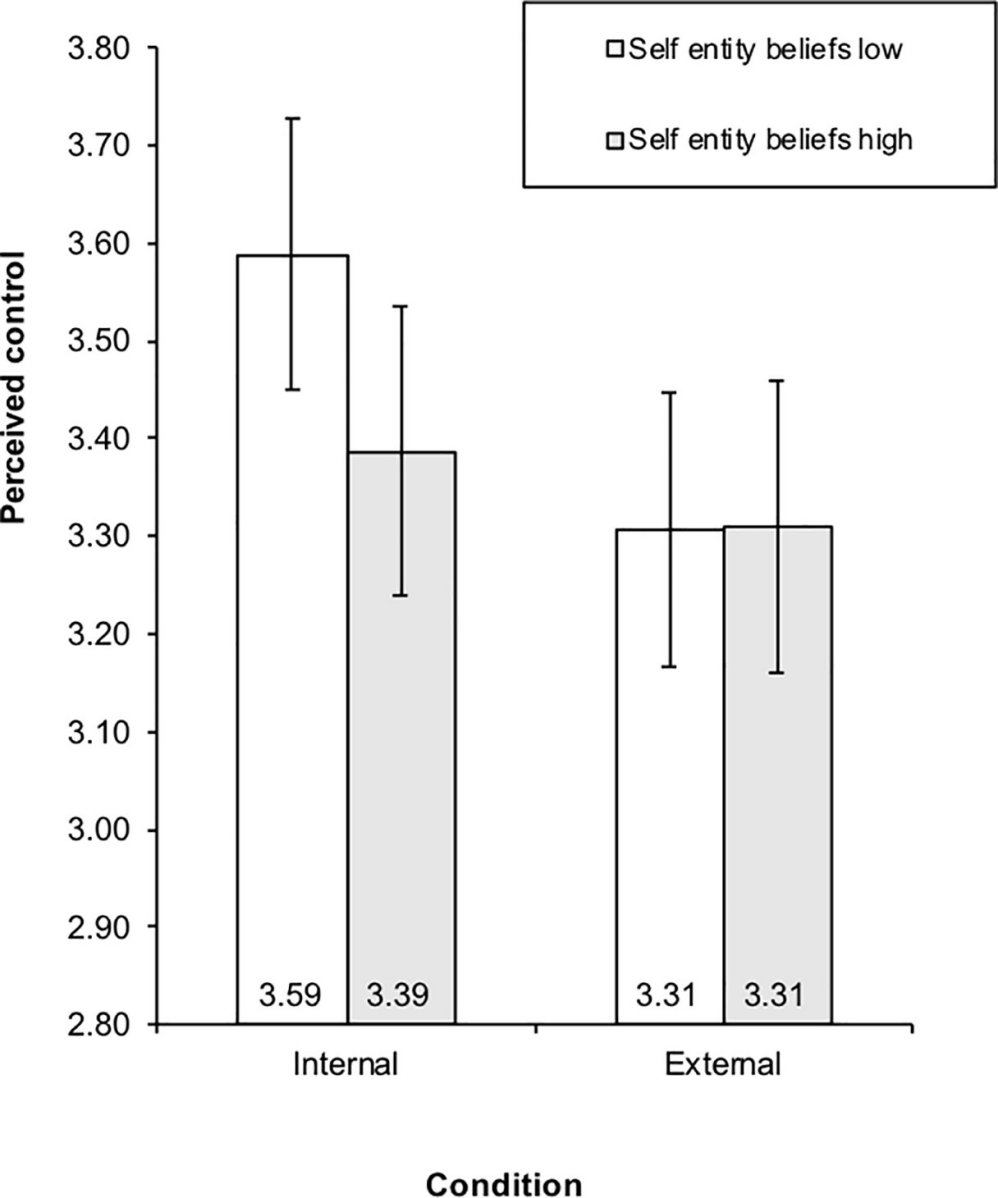
Condition by self-relevant entity beliefs interaction on control attributions.

For controllability beliefs, in the self entity beliefs ANCOVA, results indicated a main effect of sex, *F*(1, 268) = 26.68, *p* < .001, partial *η*^*2*^ = .09. Means indicated that females (*M* = 3.64, *SE* = .07) were more likely than males (*M* = 3.16, *SE* = .07) to report thinking they can do something about the events in the vignette. Results also indicated a main effect of the covariate, *F*(1, 268) = 17.21, *p* < .001, partial *η*^2^ = .06. Further a significant two-way condition X self entity beliefs interaction emerged, *F*(1, 268) = 5.97, *p* < .05, partial *η*^*2*^ = .02. This interaction is displayed in Fig **1**, which also includes the 95% confidence interval surrounding each mean and used in post hoc comparisons. As shown, individuals with high versus low entity beliefs toward their own social characteristics did not differ significantly in their attributions of controllability when vignettes depicted negative friendship events as someone else’s responsibility (external condition). Not surprisingly, perceptions of controllability were relatively low for both groups in these circumstances. However, when the negative events depicted in the vignette were portrayed as the responsibility of the participants, individuals with low entity beliefs perceived significantly greater control in the internal as opposed to external conditions and perceived significantly greater controllability than individuals with high entity beliefs in this circumstance. By contrast, the shift from external to internal attributions of responsibility did not significantly affect the perceptions of controllability of high entity theorists, who perceived their control in this circumstances as similar to that within the external attribution condition.

The other-referent entity beliefs ANCOVA on controllability beliefs replicated the main effects of sex, *F*(1, 268) = 26.12, *p* < .001, partial *η*^*2*^ = .09, and the pessimism covariate, *F*(1, 268) = 17.10, *p* < .001, partial *η*^*2*^ = .06. Means indicated that females (*M* = 3.64, *SE* = .07) were more likely than males (*M* = 3.16, *SE* = .07) to report thinking they can do something about the events in the vignette.

### Entity beliefs and behavior

The influence of social entity beliefs and the interactions with condition and jealousy on behavior was examined separately for beliefs about the self and others. Each analysis used a 2 (sex) X 2 (high versus low entity; median split) X 2 (internal versus external condition) X 2 (high versus low jealousy; median split) x 3 (behavioral response category) five-way ANCOVA, controlling for the effects of general pessimistic explanatory style. In this analysis, sex, entity beliefs, and characteristic jealousy are between subject factors. Internal versus external attribution condition and behavioral response (asocial, antisocial, and prosocial) are repeated factors.

#### Self-relevant entity beliefs

The first ANCOVA using self-relevant entity beliefs revealed main effects for entity beliefs (Fig 1), *F*(1, 259) = 8.17, *p* < .01, partial η^2^ = .03, characteristic jealousy, *F*(1, 259) = 4.23, *p* < .05, partial *η*^2^ = .02, and behavior, *F*(2, 258) = 37.43, *p* < .05, partial *η*^2^ = .23. Main effects for sex, condition and the covariate were not significant. An examination of the means indicated that children holding high entity beliefs had stronger behavioral reactions of any type (*M* = 2.38, *SE* = .03) than children holding more flexible views of their personal and social characteristics (*M* = 2.25, *SE* = .03). Also, children reporting high levels of characteristic jealousy had stronger behavioral responses (*M* = 2.36, *SE* = .03) than children who reported low levels of characteristic jealousy (*M* = 2.27, *SE* = .03).

**Fig 2:**
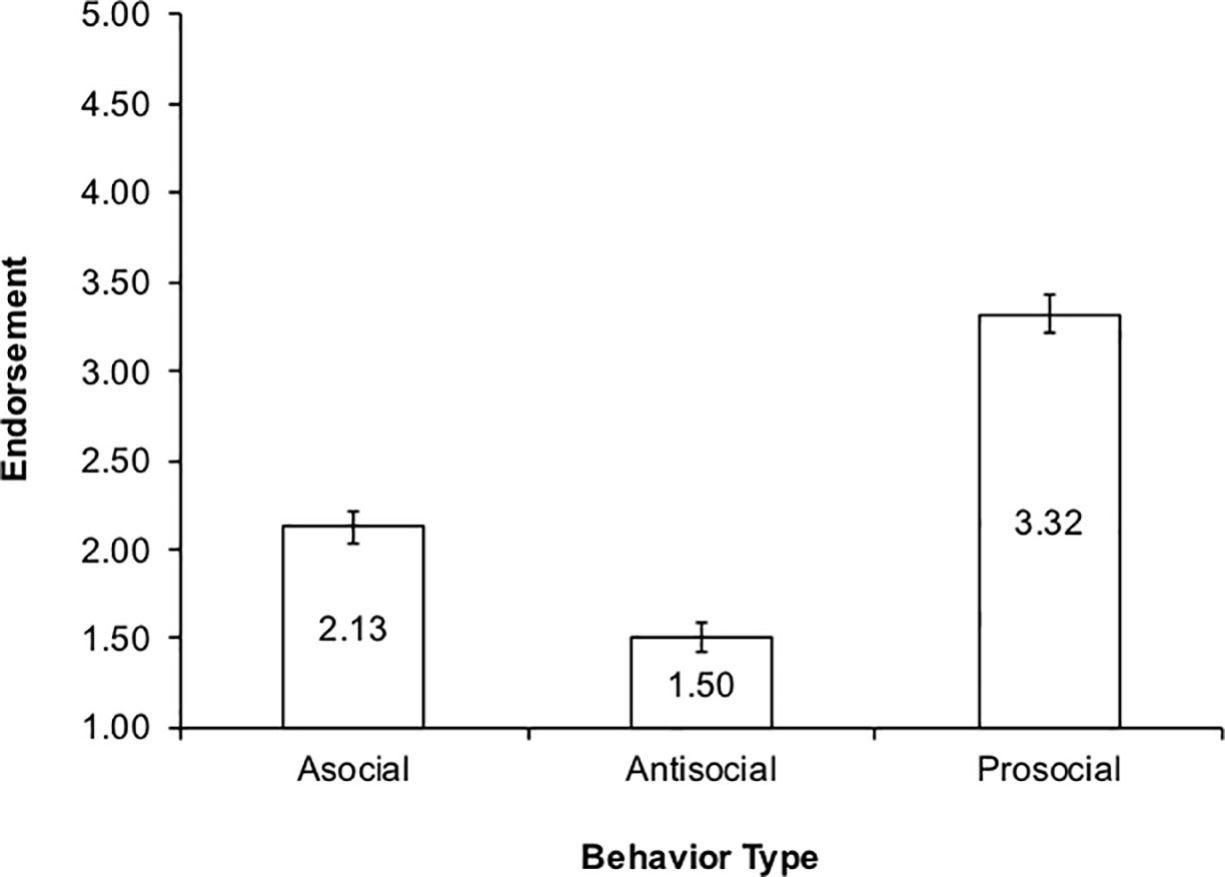
Behavior main effect.

In examining the behavior effect (Fig 2), post hoc follow-up comparisons with a Bonferroni correction (*p* < .05) and examinations of the means and confidence intervals indicated that children’s prosocial responses (M = 3.32, SE = .05) were higher than their asocial (M = 2.13, SE = .05) and antisocial (M = 1.50, SE = .04) responses. Further, their asocial responses were higher than their antisocial responses.

Results also indicated three 2-way interactions, including sex by behavior, *F*(2, 258) = 16.83, *p* < .001, partial *η*^2^ = .12, condition by behavior, *F*(2, 258) = 6.46, *p* < .01, partial η^2^ = .05, and behavior by pessimism covariate, *F*(2, 258) = 16.25, *p* < .001, partial *η*^2^ = .11. Three-way interactions included sex by behavior by jealousy, *F*(2, 258) = 3.73, *p* < .05, partial *η*^2^ = .03, and condition by behavior by pessimism covariate, *F*(2, 258) = 5.75, *p* < .01, partial *η*^2^ = .04. The hypothesized 3-way condition by behavior by self-relevant entity beliefs interaction was not significant, nor was this interaction nested within a significant 4- or 5-way interaction, and it remained non-significant when the covariate was removed.

**Fig 3:**
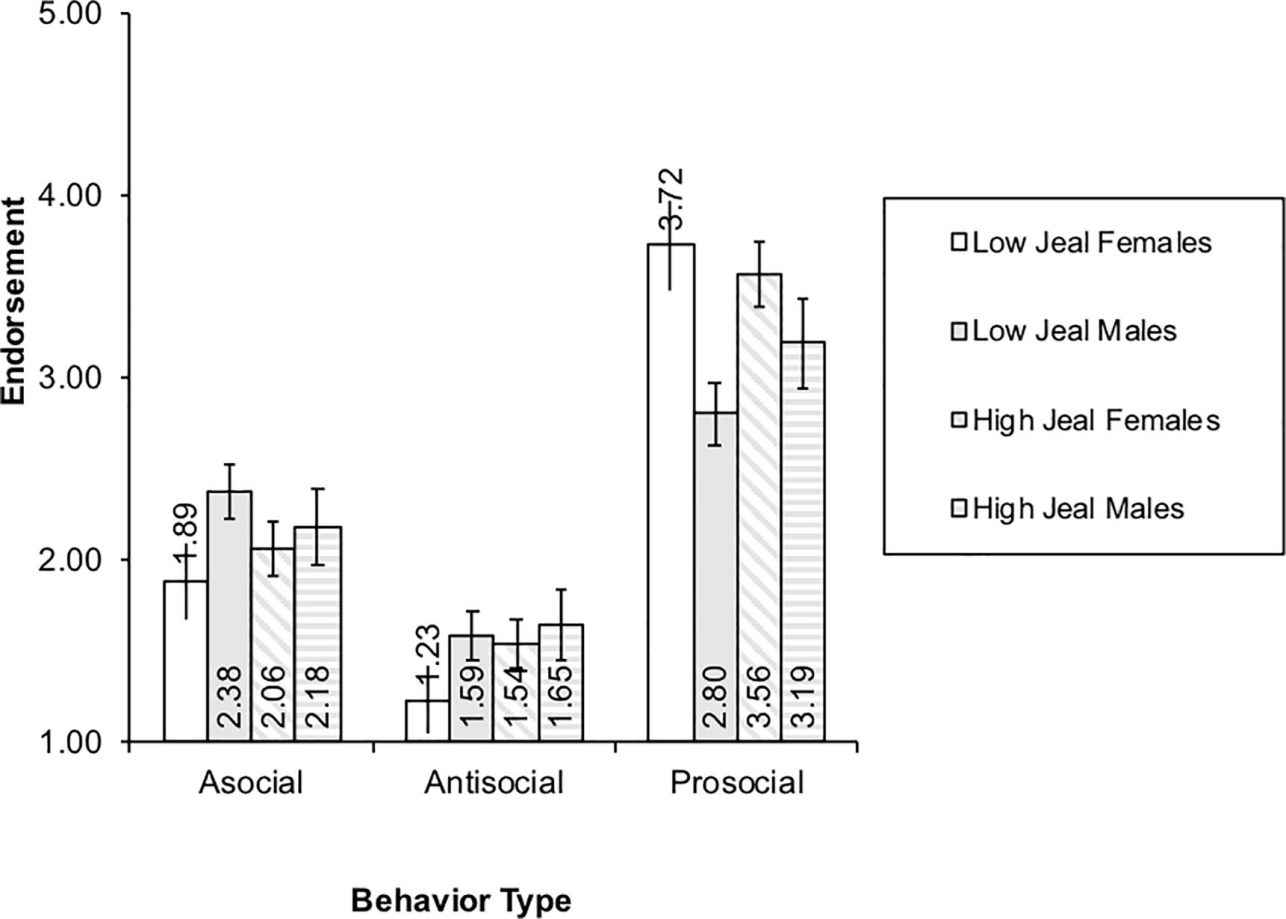
Sex by behavior by jealousy three-way interaction.

As the sex by behavior interaction was nested within the three-way sex by behavior by jealousy interaction, only the results from the three-way interaction are plotted. As Fig 3 illustrates, low-jealousy males had stronger asocial responses than females (high- or low-jealousy). Low-jealousy females had weaker antisocial responses than males. However, the strength of high-jealousy females’ antisocial responses was not significantly different from males. Finally, low-jealous females had stronger prosocial responding than all males, while low-jealous males had the weakest prosocial responses, and were significantly lower than all females.

**Fig 4:**
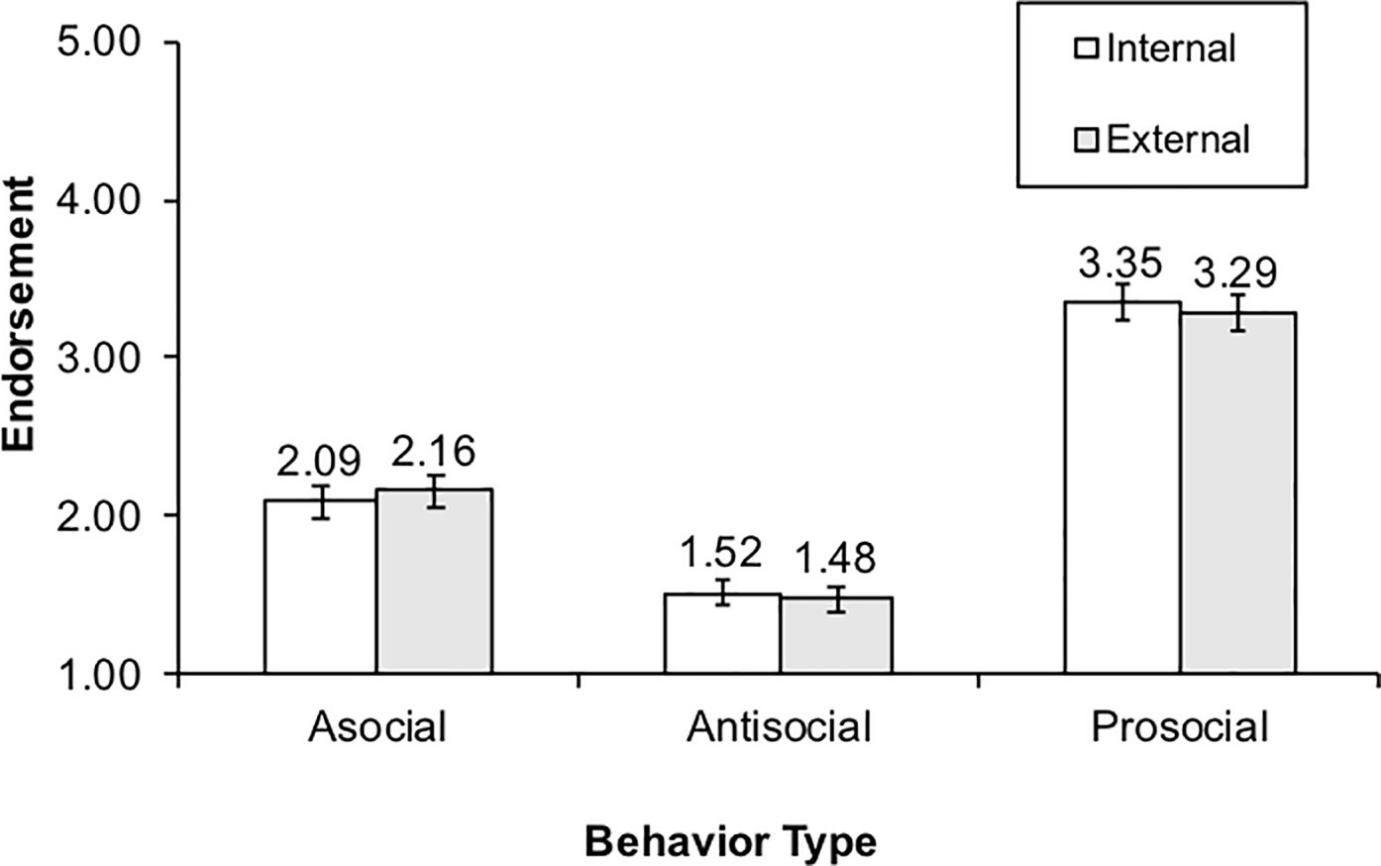
Condition by behavior two-way interaction.

Fig 4 illustrates the condition by behavior 2-way interaction. Although the effect was significant, an examination of the means and confidence intervals indicates no significant differences between the internal and external condition on any of the three behavior types.

**Fig 5:**
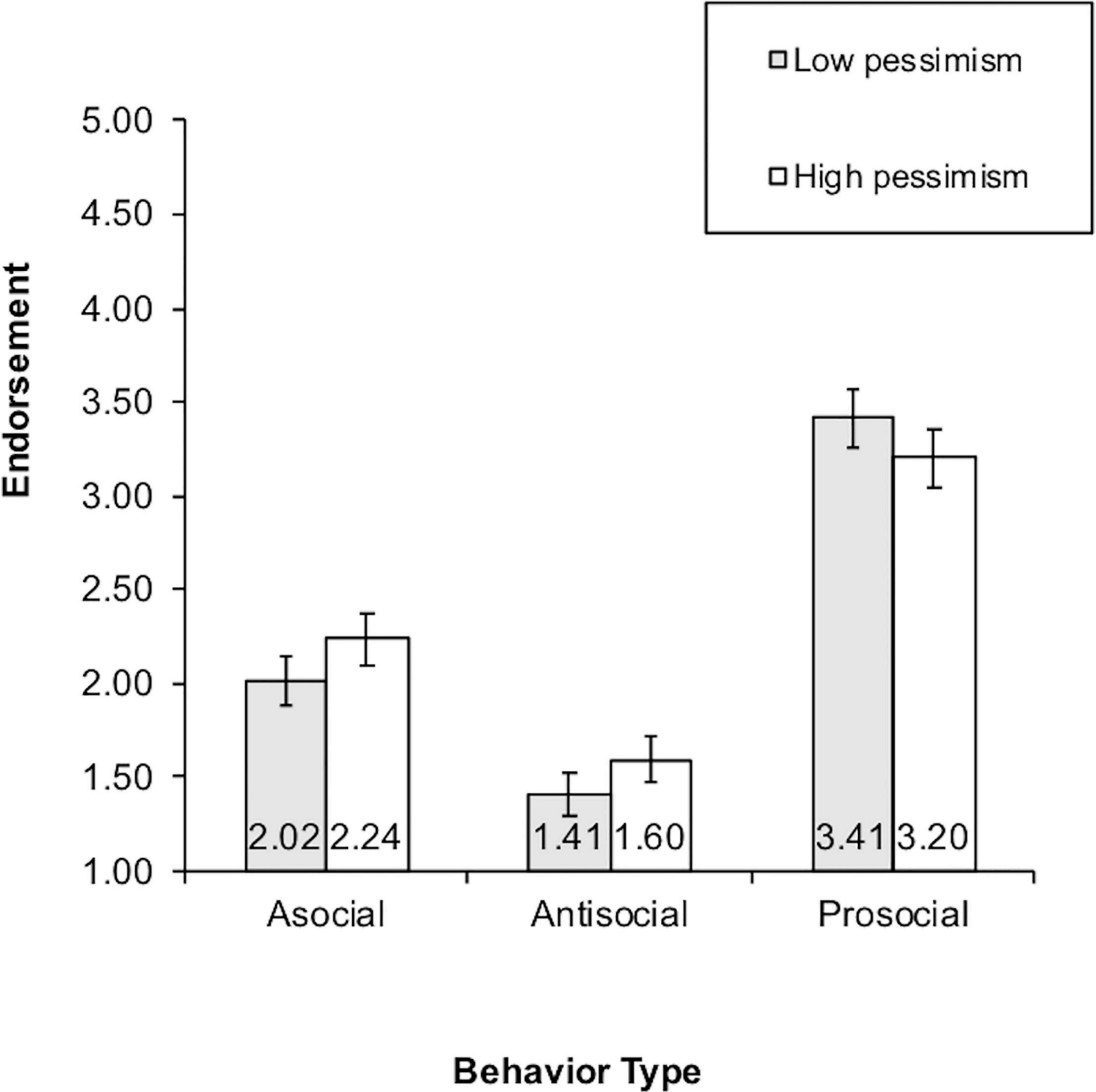
Two-way pessimism by behavior interaction.

The condition by behavior by pessimism 3-way interaction was further explored in an additional analysis to determine whether global pessimism was producing the effects hypothesized for self-relevant entity beliefs. Pessimism was dichotomized via median split and used as a factor in a 2 (sex) X 2 (high versus low pessimism) X 2 (internal versus external condition) X 2 (high versus low jealousy) X 3 (behavior) ANOVA. In this analysis, the condition by behavior by pessimism 3-way interaction was significant only at the trend level, *F*(2, 260) = 2.41, *p* < .10, partial η^2^ = .02. However, the two-way behavior by pessimism interaction was significant, *F*(2, 260) = 2.26, *p* < .05, partial *η*^2^ = .02. It also remained significant when controlling for grade, which correlated with pessimism for boys. The results of the 2-way behavior by pessimism interaction are presented in Fig 5. The confidence intervals overlap for children in the high and low pessimism groups for each behavior. However, the data do show a tendency for pessimistic children to have stronger asocial and antisocial behavior responses than low-pessimistic children. Low pessimistic children have stronger prosocial responses than pessimistic children.

#### Other-relevant entity beliefs

The planned ANCOVA was rerun substituting entity beliefs about others’ social characteristics in place of entity beliefs about one’s own characteristics. This analysis replicated the main effect for behavior, *F*(2, 258) = 38.18, *p* < .001, partial *η*^2^ = .23, and entity beliefs, *F*(2, 258) = 4.50, *p* < .05, partial *η*^2^ = .02. This analysis also yielded a main effect of the pessimism covariate, *F*(1, 259) = 4.80, *p* < .05, partial *η*^2^ = .02. Main effects for sex, jealousy and condition were not significant. As with self-relevant entity beliefs, means indicated that children holding high entity beliefs had stronger responses to the behaviors in this study (*M* = 2.36, *SE* = .03) than children holding more flexible views of others’ personal and social characteristics (*M* = 2.26, *SE* = .03).

The present analysis also replicated the three two-way interactions between sex and behavior category, *F*(2, 258) = 15.53, *p* < .001, partial *η*^2^ = .11, condition by behavior category, *F*(2, 258) = 7.94, *p* < .01, partial *η*^2^ = .06, and behavior by pessimism covariate, *F*(2, 258) = 16.76, *p* < .001, partial *η*^2^ = .12. The direction of these effects was the same as in the self-relevant entity analyses.

Although the behavior by jealousy by sex three-way interaction was not replicated, the condition by behavior by pessimism covariate interaction was replicated, *F*(2, 258) = 5.75, *p* < .01, partial *η*^2^ = .05. As in the analysis with self-relevant entity beliefs, the hypothesized 3-way condition by behavior by other entity beliefs interaction was not significant, nor was this interaction nested within a significant 4-way or 5-way interaction, and remained non-significant when the covariate was removed.

### Post-hoc analyses

Several post hoc analyses were undertaken to rule out possible alternative explanations for the absent primary hypothesized finding (specifically, the condition by entity beliefs by behavior 3-way interaction).

#### Entity beliefs composite

First, self-relevant and other-relevant entity beliefs items were composited to yield a single 6-item entity beliefs scale. Though the alpha for this scale (.79) was higher than for the two 3-item scales, using the composite scale in the 5-way ANCOVA on behavior did yield any additional significant effects.

#### Accurate condition perception

Second, the possibility that poor manipulation effectiveness attenuated effects on attributions and behavior was explored by repeating the ANCOVAs including only participants who correctly answered the manipulation check after each vignette (N = 145). The ANCOVAs on controllability attributions did not reveal any new significant effects.

The 5-way ANCOVA on behavior with self-relevant entity beliefs replicated a single main effects for behavior, *F*(2, 135) = 10.92, *p* < .001, partial *η*^2^ = .14. Main effects for sex, entity beliefs, jealousy, condition and the covariate were not significant. Results also indicated three 2-way interactions, including sex by behavior, *F*(2, 135) = 8.35, *p* < .001, partial *η*^2^ = .11, condition by behavior, *F*(2, 135) = 3.35, *p* < .05, partial η^2^ = .05, and behavior by pessimism covariate, *F*(2, 135) = 4.46, *p* < .001, partial *η*^2^ = .06. Three-way interactions included condition by behavior by jealousy, *F*(2, 135) = 3.84, *p* < .05, partial *η*^2^ = .05, and condition by behavior by self-relevant social entity beliefs, *F*(2, 135) = 4.25, *p* < .05, partial *η*^2^ = .06. No significant 4- or 5-way interactions emerged. Only the hypothesized 3-way interaction was further examined.

**Fig 6:**
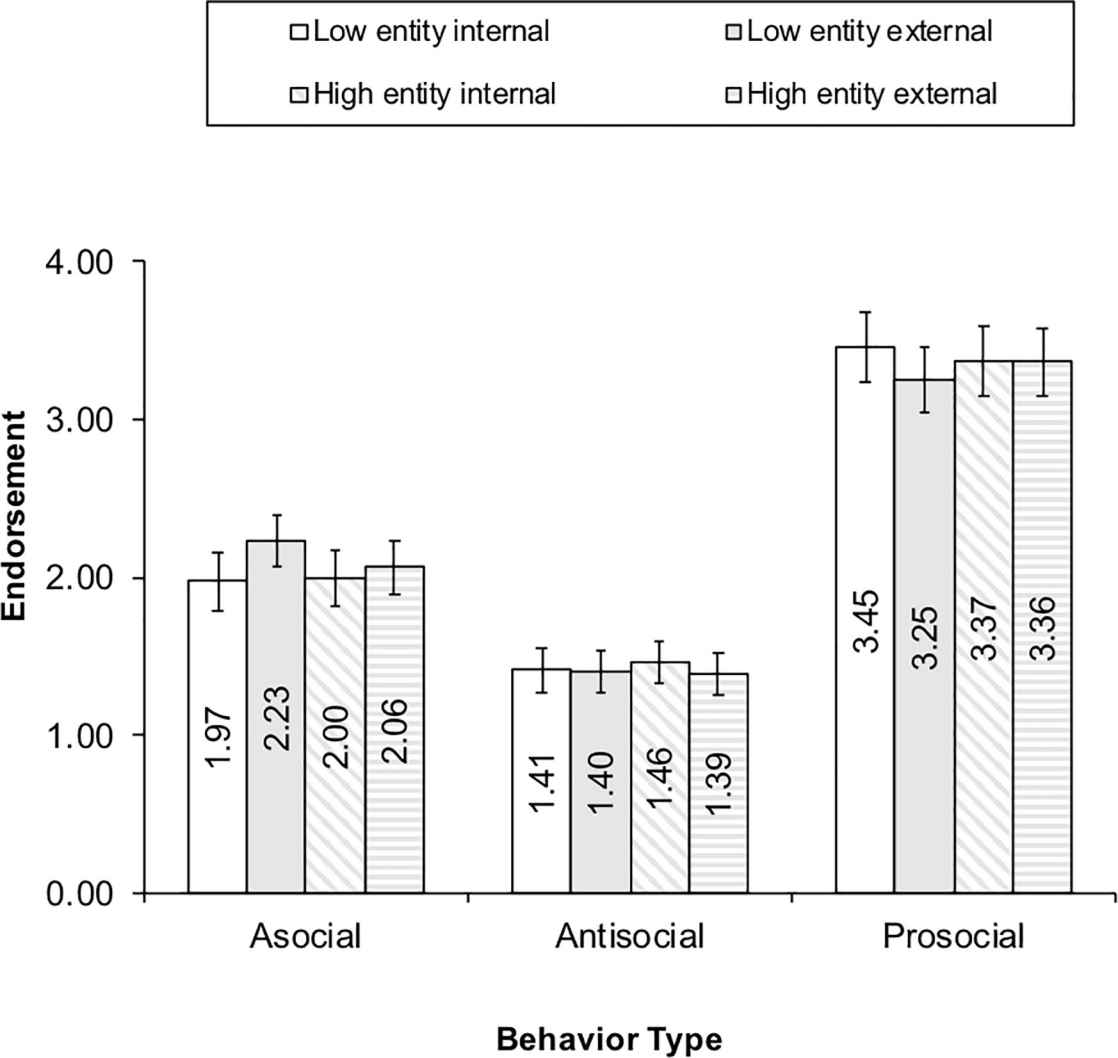
Three-way self-relevant entity by condition by behavior interaction.

Fig 6 illustrates the hypothesized self-relevant entity by condition by behavior 3-way interaction. The confidence intervals overlap for children in the high and low entity group and in each condition for each behavior. However, the data do show a tendency for low entity children in the external condition to more strongly endorse asocial behavior than children in the external condition and holding high entity beliefs. Further, the low entity children seemed to give slightly stronger endorsements to prosocial behaviors when in the internal condition than in the external condition.

The planned ANCOVA on behavior was rerun substituting entity beliefs about others’ social characteristics in place of entity beliefs about one’s own characteristics. As with the self-relevant ANCOVA, this analysis replicated the main effect for behavior, *F*(2, 135) = 10.89, *p* < .001, partial *η*^2^ = .14. Main effects for sex, jealousy, entity beliefs, condition, and the covariate were not significant. The present analysis also replicated the three two-way interactions between sex and behavior category, *F*(2, 135) = 10.62, *p* < .001, partial *η*^2^ = .14, condition by behavior category, *F*(2, 135) = 3.92, *p* < .05, partial *η*^2^ = .06, and behavior by pessimism covariate, *F*(2, 135) = 4.39, *p* < .05 partial *η*^2^ = .06. The direction of these effects was the same as in the self-relevant entity analyses.

Three-way interactions included the condition by behavior by jealousy interaction, *F*(2, 135) = 3.11, *p* < .05, partial *η*^2^ = .04. Two other significant 3-way interactions were also significant, including the condition by behavior by covariate interaction, *F*(2, 135) = 3.22, *p* < .05, partial *η*^2^ = .05, and entity by jealousy by sex, *F*(1, 136) = 4.30, *p* < .05, partial *η*^2^ = .03. The condition by behavior by entity beliefs interaction was not replicated for other-relevant beliefs.

#### Correlational analyses

The possibility that the arbitrary cut-off and reduced variance induced by the median split on entity beliefs reduced the measure’s effectiveness as a predictor was examined in a post hoc correlational analysis. Bivariate correlations between entity beliefs and the three behavior types indicate significant or trend-level positive correlations between entity beliefs and asocial behavior for both self-relevant (r = .18, p < .01 in both the internal and external conditions) and other-relevant (r = .16, p < .05 for the internal condition, r = .11, p < .10 for the external condition). Bivariate correlations between entity beliefs and antisocial and prosocial behavior were not significant.

Correlations between pessimism and behavior were also examined. Bivariate correlations between pessimism and asocial behavior are positive and moderate (.31 for internal, .20 for external, p < .01). Similarly, the correlations between pessimism and antisocial behavior are also positive (.33 for internal, .27 for external, p < .01) Pessimism is negatively correlated with prosocial behavior (-.29 for internal, -.21 for external, p < .01).

## Discussion

All children must, at some point, cope with the interference of others in their friendships. Children’s feelings of jealousy in reaction to perceived threats vary greatly and can lead to conflict with friends, loneliness and depression [2–4, 33, 34] Behavioral responses also vary widely. The present study posited that certain cognitions about social characteristics and beliefs about the controllability of the social environment would better explain behavioral responses to friendship interference than jealous emotions alone. Results indicated that views of relationships characteristics and control did enter into the picture, although not as strongly as predicted. Although entity beliefs were significantly positively related to general pessimism for girls, some of the predicted relationships involving entity beliefs were unsupported. Interestingly, some of the relationships predicted for entity beliefs were found for more general pessimism.

The analysis of controllability beliefs revealed that females were more likely than males to report thinking they can do something about friendship interference. The pessimism covariate emerged as a significant predictor. However, because an expected effect of entity beliefs emerged, the covariate effect was not reported. For self-relevant entity beliefs (but not for other-relevant entity beliefs) in the internal condition, perceptions of control were higher for children with low entity beliefs than for children with a more fixed view of social characteristics. All children seemed to agree that they had less control in the external situations than in the internal situations. Though the effect size is small, these findings are consistent with expectations.

In the prediction of behavior, both entity beliefs (both self- and other-relevant) and characteristic jealousy (in the self-relevant entity beliefs ANOVA only) predicted general endorsement of behavior. Characteristically jealous children were more likely than less jealous children to offer behavior responses in general, perhaps more motivated by their intense emotions. Unexpectedly, high endorsement of entity beliefs was also related to greater behavioral responding, in general, perhaps because there was such a high ratio of counterproductive (antisocial and asocial) behaviors relative to prosocial behaviors. However, the entity by behavior interaction was nonsignificant. Overall, the largest effect was the main effect of behavior category. Children endorsed prosocial responses the most and antisocial the least. Interactions indicated low-jealousy males are more likely than females (high- or low-jealousy) to endorse asocial behaviors and least likely to endorse prosocial behavior. Their greater willingness to withdraw from the relationship or to endorse dismissal is interesting. Perhaps because they do not see the interference as problematic, these males simply dismiss the problem altogether and do not think about it. Interestingly, low-jealousy females were less likely than males to endorse antisocial behavior and more likely to endorse prosocial behavior, yet, the experience of jealousy seemed to motivate high-jealous females to engage in higher levels of antisocial behavior, rivaling the males’ levels. High-jealousy females were just as likely as males to endorse such behavior, seemingly motivated toward aggression by their increased distress. Meta-analyses of prior research indicate that females are generally more prosocial than males, manifesting in greater altruism [35, 36], honesty [37], and cooperation [38]. Present results suggest that a lack of jealousy may be a sign of low relationship valuing and thus results in even lower prosocial behavior for males, while low jealousy actually enables females to engage in more prosocial behaviors without being clouded by negative emotions that inspire more hostile behaviors. The differential effects of jealousy on prosocial behavior in males and females should be explored further.

The hypothesized impact of entity beliefs and condition on which behaviors children endorsed received little support. However, when the analyses were re-run post hoc on only children who responded correctly to the experimental condition manipulation, the 3-way entity by condition by behavior interaction did reach significance for self-relevant entity beliefs. Although the confidence intervals overlapped for children in the high and low entity group and in each condition for each behavior, the data did show two trends. First, the data showed a tendency for low entity children in the external condition to more strongly endorse asocial behavior than children in the external condition and holding high entity beliefs. This was exactly counter to hypothesis. High entity children in the internal condition were expected to strongly endorse asocial behavior. Second, the low entity children seemed to give slightly stronger endorsements to prosocial behaviors when in the internal condition than in the external condition. Low and high entity children did not appear to differ from each other in prosocial behavior. Again, this was inconsistent with the hypothesis. Low entity children were expected to more strongly endorse prosocial behavior than high entity children, regardless of condition.

There are a number of potential reasons for the lack of expected effects, as well as limitations to this study. First, the reliability of the entity measures was not optimal. Second, the self-reported behaviors were not as accurate or objective as observations or teacher-ratings of behavior might have been. Children may exaggerate their tendency to engage in prosocial behavior when self-reporting. Third, the arbitrary cut-off and reduced variance induced by the median split on entity beliefs reduced the measure’s effectiveness as a predictor. Fourth, the social entity beliefs construct may be too specific. Perhaps the specific beliefs are idiosyncratic and general pessimistic explanatory style captures more of participants’ day-to-day worldview, providing a more reliable measure of children’s thinking in specific friendship interference situations. Finally, the true importance of control beliefs may rest in the dyad rather than the individual. That is, controllability of the specific relationship itself, may be more relevant than one’s views of relationships in general.

In conclusion, this study demonstrated that the subjective experience of jealousy does not provide a complete explanation of behavioral responding to friendship interference. Believing that social characteristics are static versus malleable did have an impact on children’s perceptions of control, yet had only a minimum effect on their endorsement of various behavioral strategies. Post hoc correlations demonstrated slightly elevated asocial responding in children which higher entity beliefs, yet results from the planned ANOVA’s did not produce significant effects. The construct of general pessimism, originally intended as a covariate, actually provided many of the effects on behavior hypothesized for social entity beliefs, contributing to increased asocial and antisocial responding and decreased prosocial responding.

## Supporting information

S1 File Manuscript data. (SAV)
